# Editorial: Microbiota in tumors: is it a new hope for treatment?

**DOI:** 10.3389/fcimb.2025.1628182

**Published:** 2025-06-20

**Authors:** Fabio Cumbo, Elena Niccolai

**Affiliations:** ^1^ Center for Computational Life Sciences, Lerner Research Institute, Cleveland Clinic, Cleveland, OH, United States; ^2^ Department of Experimental and Clinical Medicine, University of Florence, Florence, Italy

**Keywords:** intratumoral microbiome, treatment, tumor progression, editorial, microbiome

In recent years, the number of studies that link the microbiome with human health and disease has increased dramatically. The human microbiota has been increasingly recognized as a dynamic and influential component of the host immune system, metabolism, and even cancer biology. Emerging evidence has revealed that alterations in microbial composition can significantly influence the onset of tumors, as well as their progression, and response to specific therapies. These findings pushed the human microbiome into the spotlight as a promising tool for translational cancer research.

One of the most controversial developments in this regard is the potential role of intratumoral microbiota. Recent studies suggest that microbes may be actively involved in shaping the tumor’s microenvironment, with implications in creating immunosuppressive niches, driving resistance to therapies, and modulating inflammation.

Although the presence and functional relevance of these intratumoral communities remain subjects of active debates, the human microbiome continues to be a focus of intense research for its systemic effects on cancer therapy, with positive responses specifically to chemotherapy, immunotherapy, and radiation. Moreover, specific microbial signatures have been proposed as predictive biomarkers of treatment efficacy and toxicity, raising hopes for microbiome-informed precision oncology.

This Research Topic, *Microbiota in Tumors: Is it a New Hope for Treatment?*, was launched to capture the state-of-the-art in this rapidly evolving field. It brings together a range of original research studies and reviews that collectively explore the diverse roles of intratumoral microbiota in cancer biology.

Here, we highlight the five contributions published in this Research Topic, which together illustrate the diversity and novelty of current research at the intersection of microbiota and cancer.

The research study by Hu et al. investigates whether specific oral microbiota are causally linked to the risk of esophageal cancer (EC). Authors performed a two-sample Mendelian randomization (MR) approach in a cohort of 3,117 oral microbiome samples aiming at analyzing genetic variants associated with the abundance of different microbial taxa, and investigating their potential causal relationship with EC. With their analysis, authors identified 73 oral microbial taxa with statistically significant causal associations with EC, including 38 considered protective. Notably, positive associations were enriched in three phyla: *Firmicutes* (29 species), *Patescibacteria* (18 species), and *Actinobacteria* (9 species). Some taxa, including *Parvimonas micra*, *Aggregatibacter*, and *Clostridia*, showed a negative causal relationship, suggesting that EC may lead to a reduced abundance of these bacteria.

In their research study, Zhang et al. evaluate the effectiveness of the Aptima HPV E6/E7 mRNA (AHPV) assay compared to traditional liquid-based cytology (LBC) in cervical cancer screening across different age groups. Conducted between April 2018 and December 2021, the research involved female participants from 34 communities in Liaoning province and Qingdao City, China. The AHPV assay demonstrated higher sensitivity in detecting high-grade squamous intraepithelial lesions or worse (HSIL+) among women aged 25–34 compared to LBC. For women aged 35 and above, the AHPV assay showed improved specificity, reducing false-positive rates and unnecessary colposcopy referrals. These findings suggest that age-specific application of the AHPV assay, especially when combined with target genotyping, can optimize cervical cancer screening strategies, improving detection rates and reducing unnecessary procedures.

The study by Zhou et al. investigates the intratumoral microbiota landscape in human papillomavirus-independent endocervical adenocarcinoma (HPVI ECA), a rare but aggressive cervical cancer subtype. Analyzing paraffin-embedded tumor and adjacent noncancerous tissues from 45 patients via 5R-16S rDNA MiSeq sequencing, the authors found distinct microbial profiles between HPVI ECA subtypes. While overall species richness did not differ significantly between tumor and para-tumor tissues, gastric-type ECA (GEA) showed higher microbial richness than clear cell carcinoma (CCC). Importantly, the study identified specific microbial signatures associated with pathological features and prognosis. For instance, *Micrococcus* abundance was linked to advanced clinical stage and poorer patient outcomes. Random forest models revealed microbial signatures that could distinguish tumor from non-tumor tissue and differentiate HPVI ECA subtypes, underscoring the potential of microbiota as diagnostic and prognostic biomarkers in HPV-independent cervical cancer.

In the review article by Xu et al., the authors provide a comprehensive overview of how intratumoral microbiota shape the immune microenvironment of malignant tumors and affect immunotherapy outcomes. They synthesize evidence showing that microbes from various body sites influence tumor progression and therapeutic responses. These microbes modulate immune infiltration, promote immunosuppressive populations (e.g., T regulatory and myeloid-derived suppressor cells), and contribute to barrier dysfunction and metastasis. Pathogenic species like *Fusobacterium nucleatum* and *Bacteroides fragilis* are associated with immune evasion and inflammation-driven tumorigenesis, while beneficial strains like *Lactobacillus reuteri* enhance immune checkpoint inhibitor efficacy through metabolic signaling. The review also highlights engineered bacteria as emerging tools for targeted drug delivery and immune modulation. Overall, the study emphasizes that intratumoral microbiota are active modulators of tumor immunity, underscoring the importance of microbial profiling for personalized cancer diagnostics and immunotherapy.

Finally, with their opinion article, Liu and Cao discuss the emerging role of *Lactobacillus iners* within cervical tumors. Based on recent findings, the authors highlight that the presence of *L. iners* in cervical cancer tissues correlates with poorer recurrence-free survival, even in early-stage tumors. This association is attributed to the bacterium’s production of L-lactate, which reprograms tumor metabolism and promotes resistance to standard treatments like radiotherapy and chemotherapy. Additionally, *L. iners* influences key signaling pathways, including HIF-1, FGFR, Her2/ERBB2, and p53/p73, which are connected to tumor metabolism and survival. The authors propose that targeting *L. iners* via different strategies like antibiotics, metabolic inhibitors, probiotics, or synthetic biology approaches, could enhance the efficacy of tumor treatments. They conclude their article emphasizing the need for standardized methods to detect and validate *L. iners* as a reliable prognostic biomarker and therapeutic target in cervical cancer.

These studies highlight the growing recognition of the microbiome as a key player in cancer development, progression, and therapy response. Although important questions still remain unanswered, ongoing research continues to advance on the right path towards innovative diagnostic and therapeutic opportunities by uncovering tumor-specific microbial signatures, elucidating their immunomodulatory roles within the tumor microenvironment, and exploring engineered microbial strategies to enhance treatment efficacy.

